# Time gap between the onset and diagnosis in Werner syndrome: a nationwide survey and the 2020 registry in Japan

**DOI:** 10.18632/aging.202441

**Published:** 2020-12-29

**Authors:** Masaya Koshizaka, Yoshiro Maezawa, Yukari Maeda, Mayumi Shoji, Hisaya Kato, Hiyori Kaneko, Takahiro Ishikawa, Daisuke Kinoshita, Kazuki Kobayashi, Junji Kawashima, Akiko Sekiguchi, Sei-ichiro Motegi, Hironori Nakagami, Yoshihiko Yamada, Shinji Tsukamoto, Akira Taniguchi, Ken Sugimoto, Yukiko Shoda, Kunihiko Hashimoto, Toru Yoshimura, Daisuke Suzuki, Masafumi Kuzuya, Minoru Takemoto, Koutaro Yokote

**Affiliations:** 1 Department of Endocrinology, Hematology, and Gerontology, Chiba University Graduate School of Medicine, Chiba, Japan; 2 Department of Diabetes and Metabolism, Asahi General Hospital, Asahi, Chiba, Japan; 3 Department of Metabolic Medicine, Faculty of Life Sciences, Kumamoto University, Kumamoto, Japan; 4 Department of Dermatology, Gunma University Graduate School of Medicine, Maebashi, Japan; 5 Department of Health Development and Medicine, Osaka University Graduate School of Medicine, Osaka, Japan; 6 Department of Medicine, Division of Diabetes, Metabolism and Endocrinology, Atami Hospital, International University of Health and Welfare, Atami, Japan; 7 Department of Orthopaedic Surgery, Nara Medical University, Nara, Japan; 8 Geriatric and General Medicine, Osaka University, Osaka, Japan; 9 Department of Dermatology, Sumitomo Hospital, Osaka, Japan; 10 Department of Diabetes and Endocrinology, Daini Osaka Police Hospital, Osaka, Japan; 11 Diabetes and Endocrinology, Saga-Ken Medical Centre Koseikan, Saga, Japan; 12 Department of Dermatology, Showa General Hospital, Tokyo, Japan; 13 Department of Community Healthcare and Geriatrics, Nagoya University Graduate School of Medicine, Nagoya, Japan; 14 Department of Medicine, Division of Diabetes, Metabolism and Endocrinology, International University of Health and Welfare, Narita, Japan

**Keywords:** Werner syndrome, registry, nationwide survey, premature aging, sarcopenia

## Abstract

Patients with Werner syndrome present with diverse signs of aging that begin in adolescence. A Japanese nationwide survey was conducted to establish a registry that could clarify the disease profile of patients with Werner syndrome. The questionnaires were sent to 7888 doctors. The survey identified 116 patients diagnosed with Werner syndrome based on the diagnosis criteria. Forty patients were enrolled in the registry. Data on clinical symptoms, treatment information, and laboratory examination from patients who provided informed consent were collected. The data at enrollment were analyzed. The patients’ average age at enrollment was 50.1±7.5 years. The mean onset age was 26.1±9.5 years, but the mean age at diagnosis was 42.5±8.6 years. Average height and weight of the study patients were lower than those of Japanese individuals. Almost all patients experienced hair change and cataracts. More than 60% of patients presented with glycolipid abnormalities. Overall, 15% of patients had a history of foot amputation. Approximately 30% of the patients’ parents had a consanguineous marriage. The average grip strength, walking speed, and skeletal muscle mass index met the diagnostic criteria for sarcopenia. The registry revealed that there are opportunities for early diagnosis and intervention; therefore, sensitization about the disease is needed.

## INTRODUCTION

Werner syndrome is a rare autosomal recessive, adult-onset progeroid syndromes resulting from genetic instability [1]. Although the exact number of patients diagnosed with Werner syndrome in Japan is unknown, it is estimated that there are approximately 2000 patients in Japan [2–5]. Patients with Werner syndrome display various signs of aging that appear from the second decade of life. Gray hair and hair loss appear around 20 years of age, bilateral cataracts and diabetes mellitus appear at 30 years of age, and myocardial infarctions and malignant tumors appear at 40 years. Patients with Werner syndrome die around the fifth decade of life [6]. A high percentage of patients also have intractable skin ulcers [7], which can lead to amputation of the lower limbs. Characteristics of patients with Werner syndrome include having a bird-like face and a high-pitched voice, which may offer difficulties with social integration. Sensitization of patients and medical practitioners on Werner syndrome is required to improve the quality of medical treatment, support the social reintegration, and improve the prognosis of patients with Werner syndrome.

Furthermore, manifestations of the disease can vary widely by individual with different grades of severity and age of onset. Coupled with an overall disease rarity, this heterogeneity makes diagnosis difficult and requires clearer guidelines.

Therefore, this study aimed to reveal the current disease profile of patients with Werner syndrome in Japan by conducting a nationwide survey and through the establishment of the Werner Syndrome Registry.

## RESULTS

### Werner syndrome nationwide survey in Japan

A nationwide survey of Werner syndrome was conducted with the goal of creating a Japanese Werner syndrome registry. In 2017, questionnaires were sent to 7888 doctors affiliated with hospitals that have more than 200 beds, and who work in divisions of internal medicine (endocrinology, collagen disease, and geriatrics), ophthalmology, dermatology, plastic surgery, or orthopedic specialties. Of the questionnaires sent out, 3154 (40%) responses were received. A total of 116 patients (57 men and 59 women) were being treated at the hospitals at the time of the survey (Supplementary Table 1). Fifty-one patients (29 men, 22 women) were suspected of having Werner syndrome. In addition, although they had not been attending the hospitals during the survey, there were 153 patients, including 80 men and 71 women (the sexes of two patients were unknown), who had visited the hospital in the past 10 years.

The breakdown based on the departments that responded to the survey is shown in Table 1. The percentage of reported patients per researched clinical departments is also presented. Ninety-seven patients were reported from the departments of internal medicine, which included metabolism and geriatric medicine. However, the departments of plastic surgery and dermatology reported the highest proportion of patients (7.7% and 7.9%). Patient overwraps could not be excluded completely. Two clinical departments answered in 32 facilities. Three clinical departments answered in 6 facilities.

**Table 1:** Number of patients with Werner syndrome by attendance to different clinical department.

	**Reported patients (n)**	**Researched clinical departments (n)**	**Reported patients / researched clinical departments (%)**
Internal medicine	97	2804	3.5
Dermatology	91	1147	7.9
Plastic surgery	57	744	7.7
Orthopedics	40	1319	3.0
Ophthalmology	28	1165	2.4
Cardiac surgery	7	713	1.0
Total	320	7892	4.1

Reported patients includes 116 diagnosed patients who were attending the hospital for treatment during the survey, 51 patients suspected of having Werner syndrome, and 153 patients visited the hospital in the past 10 years although not having attended the hospitals during the survey. The numerator is "reported patients" and the denominator is "researched clinical departments". Patient overwraps could not be excluded completely. Two clinical departments answered in 32 facilities. Three clinical departments answered in 6 facilities.

**Figure 1:**
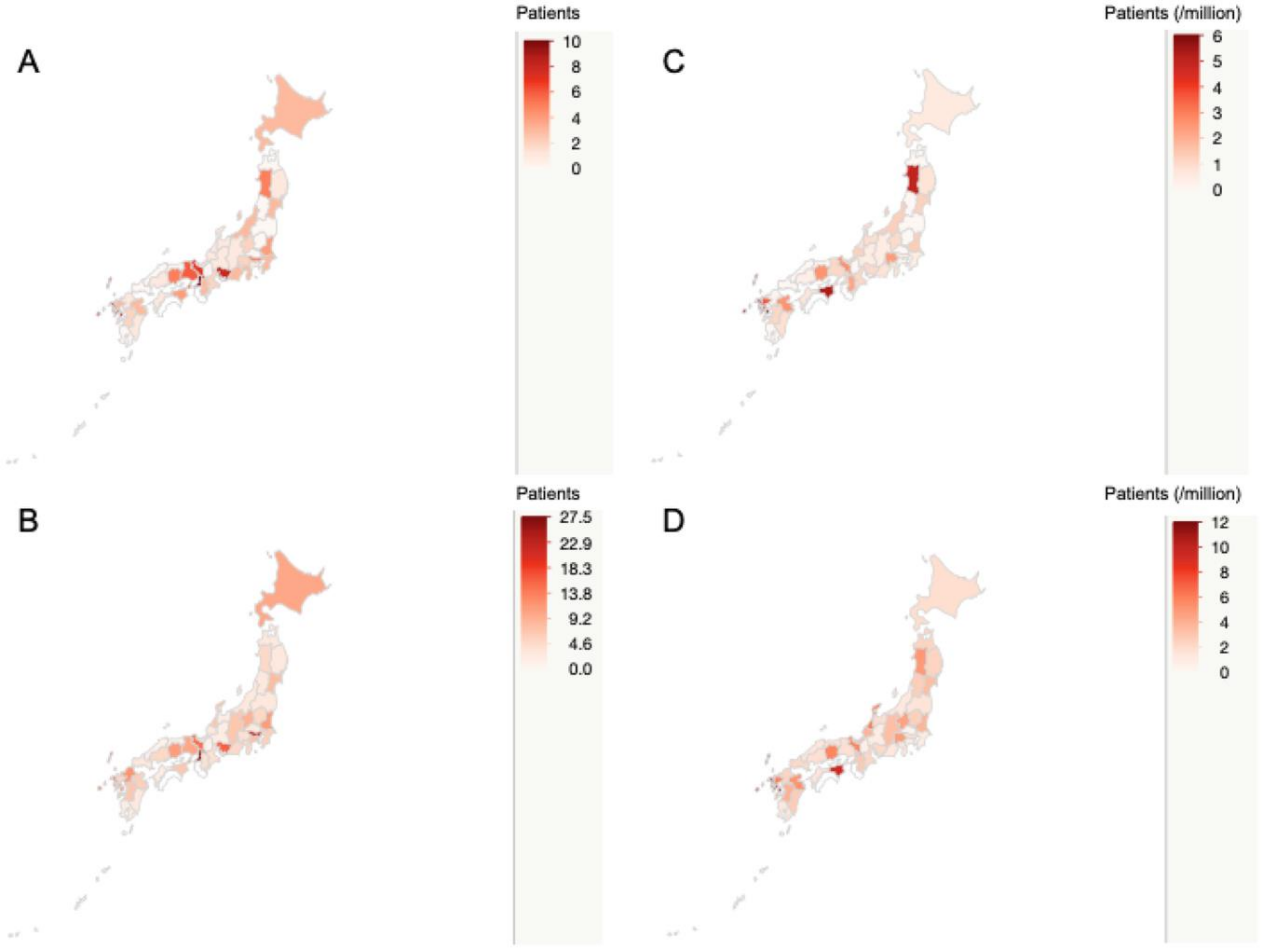
**Nationwide distribution of patients with Werner syndrome in Japan.** The map shows the nationwide distribution of patients with Werner syndrome. The concentration of red indicates the number of patients. The upper left map (**A**) shows the nationwide distribution of diagnosed cases. The lower left map (**B**) shows the nationwide distribution of total patients including diagnosed cases, suspected cases, and past confirmed cases. The upper right map (**C**) shows the diagnosed cases per million population. The lower right map (**D**) shows total patients including diagnosed cases, suspected cases, and past confirmed cases per million population.

The distribution of the patients is shown in Figure 1. Werner syndrome was distributed nationally (Figure 1A). A combination of diagnosed cases, suspected cases, and past confirmed cases was also evenly distributed throughout Japan (Figure 1B). Since there are differences in population in each region, the patient population per million people was calculated for diagnosed cases (Figure 1C), and for the combination of diagnosed cases, suspected cases, and past confirmed cases (Figure 1D). There were no statistically significant differences in the distributions of the patients with Werner syndrome (*P*=0.471). Details are shown in Supplementary Table 2 and Supplementary Figure 1.

### Werner syndrome registry cross-sectional analysis

Thirty-two facilities participated in the registry. Of the 116 diagnosed patients in the nationwide survey, 40 (34.5%) were enrolled in the registry. Table 2 shows the major signs of Werner syndrome, which include graying hair, hair loss, cataracts, skin atrophic changes, and soft-tissue calcification. Almost all patients exhibited some of the major signs. Approximately 90% of patients had a characteristic bird-like face and high-pitched voice. Over half of the patients had diabetes, impaired glucose tolerance (67.5%), dyslipidemia (65.0%), and fatty liver (52.5%). A small percentage of patients had a history of atherosclerosis, such as cerebral infarction (0%), angina pectoris or myocardial infarction (2.5%), or arteriosclerosis obliterans (ASO) (15.0%) (Table 2). Limb amputation was observed in 15.0% of patients. Malignant tumor was observed in 20.0% of patients (Table 2) and comprised lung cancer, lung adenocarcinoma, undifferentiated pleomorphic sarcoma, fibrosarcoma, osteosarcoma, colorectal cancer, follicular thyroid cancer, and melanoma.

**Table 2:** Frequency of major signs, clinical symptoms, and medications administered to patients with Werner syndrome.

	**%**	**n**	**N**
**Major signs**			
Graying of hair, hair loss	97.5	39	40
Cataracts	100	40	40
Skin changes	97.5	39	40
Intractable skin ulcers	67.5	27	40
Soft-tissue calcification	87.5	35	40
Bird-like face	90	36	40
High-pitched voice	87.5	35	40
**Clinical symptoms**			
Diabetes, IGT	67.5	27	40
Dyslipidemia	65	26	40
Hypertension	42.5	17	40
Fatty liver	52.5	21	40
Cerebral bleeding	0	0	40
Cerebral infarction	0	0	40
AP or MI	2.5	1	40
ASO	15	6	40
Amputation	15	6	40
Malignant tumor	20	8	40
**Medications**			
**Diabetes, IGT**			
DPP-4 inhibitor	37.0	10	27
Biguanide	33.3	9	27
Thiazolidine	48.1	13	27
alpha GI	7.4	2	27
Sulfonylurea	11.1	3	27
SGLT2 inhibitor	3.7	1	27
Glinide	0	0	27
GLP-1 analog	3.7	1	27
Insulin	14.8	4	27
**Dyslipidemia**			
Statin	65.4	17	26
Fibrate	3.8	1	26
Ezetimibe	0	0	26
EPA	11.5	3	26
Resin	0.0	0	26
Nicotinic acid	19.2	5	26
Probucol	0	0	26
**Hypertension, among others**			
Ca blocker	47.1	8	17
ARB	35.3	6	17
ACE inhibitor	0.0	0	17
Alpha1 blocker	0.0	0	17
Beta blocker	11.8	2	17
Diuretics	0.0	0	17
Antiplatelet	5.0	2	40
Anticoagulant	12.5	5	40

The percentages of medications administered to treat abnormal glucose metabolism, dyslipidemia, and hypertension are shown for the total number of patients with each disorder. N, number of patients; n, number of patients with symptoms or treatment drugs, IGT, Impaired glucose tolerance; AP, Angina pectoris; MI, Myocardial infarction; ASO, Arteriosclerosis obliterans; DPP-4, Dipeptidyl peptidase-4; alpha GI, alpha glucosidase inhibitor; SGLT2, Sodium-glucose cotransporter-2; GLP-1, Glucagon-like peptide-1; EPA, Eicosapentaenoic acid; Ca, Calcium; ARB, Angiotensin-II receptor blocker; ACE, Angiotensin-converting enzyme inhibitor.

Table 2 provides details of the medications administered to treat abnormal glucose metabolism, dyslipidemia, and hypertension. More than 30% of patients with diabetes were treated with a dipeptidyl peptidase-4 (DPP-4) inhibitor, biguanide, or thiazolidine. Two-thirds of patients with dyslipidemia were treated with a statin. Calcium antagonists and angiotensin-II receptor blockers were often used for patients with hypertension.

The change in major signs and clinical symptoms over time is shown in Table 3. There were no differences in patients’ demographic characteristics between 2009 and 2017; sex (male 46.6% vs. 55.0%, *P*=0.388). Although the data in 2009 had age groups and not the actual age, the mean age group in 2009 was 50s, while the mean age in 2017 was 50.1 years. Compared with that in 2009, the percentage of patients with intractable skin ulcers had decreased (87.5% vs. 67.5%, *P*<0.01) and that of soft-tissue calcification had increased (76.7% vs. 87.5%, *P*=0.048). The percentage of angina pectoris or myocardial infarction had significantly decreased (14.8% vs. 2.5%, *P*=0.049). The percentage of malignant tumor had significantly decreased (42.4% vs. 20.0%, *P*=0.010).

**Table 3:** Major signs and clinical symptom changes showing a positive percentage over time.

	**In 2009 (%)**	**In 2020 (%)**	***P* value**
Sex (male)	46.6	55.0	0.388
Graying of hair, hair loss	98.1	97.5	1.000
Cataracts	92.5	100	0.124
Skin changes	97.4	97.5	1.000
Intractable skin ulcers	87.5	67.5	<0.01
Soft-tissue calcification	76.7	87.5	0.048
Bird-like face	96.1	90.0	0.220
High-pitched voice	88.0	87.5	1.000
Diabetes, impaired glucose tolerance	71.43	67.5	0.700
Dyslipidemia	68.5	65.0	0.701
Hypertension	34.6	42.5	0.250
Fatty liver	44.2	52.5	0.330
Cerebral bleeding	1.5	0	1.000
Cerebral infarction	3.7	0	0.591
Angina pectoris or myocardial infarction	14.8	2.5	0.049
Arteriosclerosis obliterans	24.4	15.0	0.277
Malignant tumor	42.4	20.0	0.010
Consanguineous marriage	39.0	29.7	0.424

The data in this registry in 2020 was compared with the data of survey in 2009. Fisher’s exact test was used for the statistical comparison.

Table 4 shows the change in medications administered between 2009 and 2020. DPP-4 inhibitor, sodium-glucose cotransporter-2 (SGLT2) inhibitor, and glucagon-like peptide-1 (GLP-1) analog were not used in 2009. While not statistically significant, the use of alpha glucosidase inhibitor and sulfonylurea usage had decreased in 2020 compared with 2009. Statin and anti-hypertensive medication use was similar in 2020, while the use of fibrate had significantly decreased (19.2% vs. 3.8%, *P*=0.017).

**Table 4:** Medications administered changes over time.

	**In 2009 (%)**	**In 2020 (%)**	***P* value**
**Diabetes, IGT**			
DPP-4 inhibitor	0	37.0	<0.01
Biguanide	19.1	33.3	0.261
Thiazolidine	40.4	48.1	0.630
alpha GI	25.5	7.4	0.067
Sulfonylurea	29.8	11.1	0.086
SGLT2 inhibitor	0	3.7	0.375
Glinide	4.3	0	0.527
GLP-1 analog	0	3.7	1.000
Insulin	27.7	14.8	0.381
**Dyslipidemia**			
Statin	65.4	65.4	0.610
Fibrate	19.2	3.8	0.017
Ezetimibe	1.9	0	1.000
EPA	5.8	11.5	0.557
Resin	1.9	0	1.000
Nicotinic acid	1.9	19.2	0.153
Probucol	5.8	0	0.503
**Hypertension, among others**			
Ca blocker	42.1	47.1	1.000
ARB	47.4	35.3	0.510
ACE inhibitor	5.3	0.0	1.000
Alpha1 blocker	0.0	0.0	1.000
Beta blocker	5.3	11.8	1.000
Diuretics	10.5	0.0	0.487
Antiplatelet	NA	5.0	NA
Anticoagulant	NA	12.5	NA

The data in this registry in 2020 was compared with the data of survey in 2009. Fisher’s exact test was used for the statistical comparison.

The percentages of medications administered to treat abnormal glucose metabolism, dyslipidemia, and hypertension are shown for the total number of patients with each disorder. DPP-4, Dipeptidyl peptidase-4; alpha GI, alpha glucosidase inhibitor; SGLT2, Sodium-glucose cotransporter-2; GLP-1, Glucagon-like peptide-1; EPA, Eicosapentaenoic acid; Ca, Calcium; ARB, Angiotensin-II receptor blocker; ACE, Angiotensin-converting enzyme inhibitor, NA; not available.

Table 5 shows the results of blood tests. The mean red blood cell counts and hemoglobin levels in men were lower than the normal range. On average, patients had more than two times higher levels of gamma-glutamyl transpeptidase than the upper normal limit. Aspartate aminotransferase, alanine aminotransferase (ALT), lactate dehydrogenase, and triglyceride (TG) in men and ALT in women were slightly higher than normal range. Average glycated hemoglobin (HbA1c) level was less than 6.5%. Average fasting plasma glucose (FPG) level was less than 126 mg/dL. Average postprandial plasma glucose (PPG) level was less than 200 mg/dL. Low-density lipoprotein cholesterol (LDL-C) level was below 120 mg/dL. Although FPG, PPG, and HbA1c were higher than normal range, these values were lower than the treatment target values of diabetes and hyper LDL-cholesterolemia, which indicated that the levels of HbA1c, plasma glucose, and LDL-C levels were well controlled.

**Table 5:** Blood test findings.

	**Total**				**Men**				**Women**				**Normal range**
	**Mean**		**SD**	**n**	**Mean**		**SD**	**n**	**Mean**		**SD**	**n**	
WBC (/μL)	7502	±	2400	39	7663	±	2845	22	7293	±	1724	17	3300 – 8600
RBC (/μL)	424	±	64	39	423*	±	76	22	426	±	46	17	Men 435 – 555, Women 386 – 492
Hgb (g/dL)	12.6	±	2.0	39	13.0*	±	2.3	22	12.2	±	1.7	17	Men 13.7 – 16.8, Women 11.6 – 14.8
Plt (x 10^3^/μL)	28.9	±	7.9	39	27.1	±	6.6	22	31.2	±	8.9	17	15.8 – 34.8
AST (U/L)	29	±	13	40	34*	±	16	22	24	±	6	18	13 –30
ALT (U/L)	39	±	29	340	43*	±	30	22	34*	±	27	18	Men 10 – 42, Women 7 – 23
γ-GTP (U/L)	100*	±	116	38	87*	±	104	22	116*	±	131	16	Men 13 – 64, Women 9 – 32
LDH (U/L)	230*	±	181	37	255*	±	230	22	193	±	44	15	124 – 222
ALP (U/L)	313	±	176	34	311	±	141	21	317	±	229	13	106 – 322
ChE (U/L)	363	±	102	30	364	±	121	17	363	±	74	13	Men 240 – 486, Women 201 – 421
T-Bil (mg/dL)	0.5	±	0.2	34	0.5	±	0.3	20	0.5	±	0.2	14	0.4 – 1.5
TC (mg/dL)	194	±	34	33	198	±	35	21	187	±	34	12	125 – 219
TG (mg/dL)	158*	±	91	38	166*	±	87	21	148	±	98	17	35 – 149
LDL-C (mg/dL)	115	±	32	36	114	±	36	19	117	±	28	17	less than 140
HDL-C (mg/dL)	57	±	17	35	58	±	22	18	55	±	11	17	40 and more
TP (g/dL)	7.8	±	0.6	36	7.9	±	0.5	21	7.7	±	0.7	15	6.6 – 8.1
Alb (g/dL)	4.2	±	0.8	37	4.2	±	0.9	20	4.2	±	0.6	17	4.1 – 5.1
UA (mg/dL)	5.4	±	1.3	36	5.7	±	1.2	20	4.9	±	1.3	16	7.0 and less
BUN (mg/dL)	16	±	8	37	17	±	9	22	15	±	7	15	8 – 20
Cre (mg/dL)	0.8	±	1.0	39	1.0	±	1.2	22	0.5	±	0.2	17	Men 0.65 – 1.07, Women 0.46 – 0.79
Na (mEq/L)	139	±	3	37	139	±	3	21	139	±	4	16	138 – 145
K (mEq/L)	4.2	±	0.4	37	4.3	±	0.5	21	4.2	±	0.3	16	3.6 – 4.8
Cl (mEq/L)	104	±	4	36	105	±	3	20	103	±	4.5	16	101 – 108
Ca (mg/dL)	9.3	±	0.5	28	9.2	±	0.6	15	9.4	±	0.4	13	8.8 – 10.1
FPG (mg/dL)	114*	±	28	14	116*	±	35	6	112*	±	23	8	73 – 109
PPG (mg/dL)	144*	±	57	21	150*	±	49	12	136	±	69	9	less than 140
HbA1c (%)	6.4*	±	1.3	35	6.1*	±	0.8	18	6.8*	±	1.7	17	4.9 – 6.0

*shows abnormal values. WBC; white blood cell, RBC; red blood cell, Hgb; hemoglobin, Plt; platelet, AST; aspartate aminotransferase, ALT; alanine aminotransferase, γ-GTP; gamma-glutamyl transpeptidase, LDH; lactate dehydrogenase, ALP; alkaline phosphatase, ChE; cholinesterase, T-Bil; total bilirubin, TC; total cholesterol, TG; triglyceride, LDL-C; low-density lipoprotein cholesterol, HDL-C; high-density lipoprotein cholesterol, TP; total protein, Alb; albumin, UA; uric acid, BUN; blood urea nitrogen, Cre; creatinine, Na; natrium, K; potassium, Cl; chlorine, Ca; calcium, FPG; fasting plasma glucose, PPG; postprandial plasma glucose, HbA1c; glycated hemoglobin, SD; standard deviation.

The patients’ average age at enrollment was 50.1 ± 7.5 years. The average age at Werner syndrome onset was 26.1 ± 9.5 years; however, the age of diagnosis was 42.5 ± 8.6 years (Table 6).

**Table 6:** Patient background, physical findings, body composition, and physical function.

	**Total**				**Men**				**Women**			
	**Mean**		**SD**	**n**	**Mean**		**SD**	**n**	**Mean**		**SD**	**n**
**Patients’ backgrounds**												
Age (years)	50.1	±	7.5	40	49.4	±	7.6	22	50.9	±	7.5	18
Onset age (years)	26.1	±	9.5	30	28.2	±	8.5	16	23.7	±	10.2	14
Diagnosed age (years)	42.5	±	8.6	39	42.0	±	6.4	21	43.2	±	10.8	18
**Physical findings**												
Height (cm)	154.0	±	10.7	40	159.7	±	8.6	22	147.2	±	9.0	18
Body weight (kg)	44.1	±	9.5	40	49.0	±	9.3	22	38.1	±	5.4	18
BMI (kg/m^2^)	18.5	±	3.1	40	19.2	±	3.5	22	17.6	±	2.5	18
Waist circumference (cm)	77.3	±	12.0	24	80.4	±	12.2	14	73.0	±	10.8	10
Visceral fat area (cm^2^)	102.3	±	61.4	10	112.4	±	81.5	4	95.6	±	51.7	6
SMI (kg/m^2^)	4.3	±	0.8	9	4.5	±	0.9	5	4.1	±	0.6	4
**Physical function**												
Mean grip strength (right) (kg)	17.1	±	8.7	23	20.8	±	8.6	13	12.3	±	6.3	10
Mean grip strength (left) (kg)	16.0	±	7.6	23	19.5	±	7.3	13	11.4	±	5.3	10
Mean walking speed (m/sec)	0.8	±	0.6	13	0.9	±	0.6	6	0.8	±	0.6	7

BMI; body mass index, SMI; skeletal muscle mass index, SD; standard deviation.

The patients’ average height, body weight, and body mass index (BMI) (159.7 cm, 49.0 kg, BMI 19.2 kg/m^2^ in men, 147.2 cm, 38.1 kg, BMI 17.6 kg/m^2^ in women) (Table 6) were lower than those of the average Japanese individual in the fifth decade of life as reported by the Japanese Ministry of Health, Labour, and Welfare's 2018 National Health and Nutrition Survey Report (169.2 cm, 68.1 kg, BMI 23.5 kg/m^2^ in men, 156.6 cm, 55.0 kg, BMI 22.2 kg/m^2^ in women). In Werner syndrome, patients present with central obesity; the average abdominal circumference was 80.4 ± 12.2 cm in men and 73.0 ± 10.8 cm in women. The average abdominal circumference was large, although the respective BMI were low (Table 6). Namely, the patients with Werner syndrome have lipodystrophy.

The average of the total limb skeletal mass index (SMI), identified using dual-energy X-ray absorptiometry (DEXA), was 4.5 ± 0.9 kg/m^2^ for men and 4.1 ± 0.6 kg/m^2^ for women. Although one patient had four toes amputated, the other patients had not undergone amputation. Grip strengths were (right) 20.8 ± 8.6 kg and (left) 19.5 ± 7.3 kg for men, and (right) 12.3 ± 6.3 kg and (left) 11.4 ± 5.3 kg for women. Walking speed was 0.8 ± 0.6 m/sec on average (Table 6).

## DISCUSSION

Our mission is to improve the prognosis and support social reintegration for patients with Werner syndrome, by improving the quality of medical treatment. To address the clinical questions regarding Werner syndrome and to collect high quality evidence, we conducted this nationwide survey and established the Werner Syndrome Registry (case registration system). The Werner syndrome nationwide survey identified 116 confirmed cases of Werner syndrome in Japan; a total of 32 facilities participated in the Werner Syndrome Registry and 40 patients were enrolled in the registry.

As the maps show, there were no statistically significant differences in the distributions of the patients with Werner syndrome throughout Japan in 2017. However, compared to the patient population per million people for each region in 2009 (Nagasaki 7.6 patients, Tokushima 6.3 patients, Nagano 5.5 patients, Miyazaki 5.3 patients) [7], there were some changes in 2017 (Nagasaki 5.9 patients, Tokushima 5.4 patients, Akita 5.0 patients, Saga 3.6 patients), which suggests that there were regional temporal changes in the incidence of Werner syndrome.

Previously, widespread consanguineous marriages resulted in localized and uneven distribution of Werner syndrome [8]. The absence of significant regional biases in patients’ distribution across the country may be a result of the low percentage of consanguineous marriages and increased movement of the populations due to advances in transportation.

The time gap between the age of onset and the age of diagnosis was similar to that reported in the 2006 international Werner syndrome registry [9]. In the international Werner syndrome registry, the mean age of cataracts was 31 years and age of diagnosis or referral was 43 years.

These results suggest that it is necessary to consider measures for early diagnosis and early intervention. Werner syndrome onset is usually recognized by bilateral cataracts or gray hair and hair loss, which are usually the first symptoms [7]. The patients normally undergo cataract surgery around third decade of life. However, many of patients and ophthalmologists may not have adequate information to diagnose Werner syndrome. Around the fourth decade of life, the patients tend to have intractable ulcers and visit the dermatologist or plastic surgeon. As the national survey showed, many patients with Werner syndrome were reported by dermatologists or plastic surgeons, and not by ophthalmologists.

In order to promote early diagnosis of Werner syndrome, it is necessary to create awareness regarding Werner syndrome among ophthalmologists. As part of the solution, we plan to advertise in journals and conferences whose readership includes ophthalmologists.

It has previously been reported that the average life span of patients with Werner syndrome is around 50 years. However, in our analysis, the average age at enrollment was 50.1 ± 7.5 years, which suggests that the life expectancy of patients with Werner syndrome may be longer than that reported two decades ago [8]. Notably, few patients had a history of atherosclerosis, such as cerebral infarction, angina pectoris, myocardial infarction, or ASO in the registry. Compared with the previous survey conducted in 2009 [7], the percentages of patients with a history of cerebral bleeding, cerebral infarction, angina pectoris, myocardial infarction, and ASO decreased in 2020. This may have been due to improved control of diabetes with better treatment modalities. The high percentage of pioglitazone use, which increases insulin sensitivity, is a characteristic diabetes treatment for patients with Werner syndrome [10]. In the current decade, DPP-4 inhibitors are often used to treat the common form of type 2 diabetes in Japan [11]. The effectiveness of the DPP-4 inhibitor, sitagliptin, for a pioglitazone non-responder patient with Werner syndrome has been reported [12]. Reportedly, GLP-1 analog improves vascular function and reduces abdominal fat accumulation in patients with Werner syndrome [13]. In a large-scale clinical study for type 2 diabetes, the cardiovascular preventive effects of GLP-1 analog and SGLT2 inhibitor have been reported [14–18].

Although the usage of fibrate decreased, there is little evidence that fibrate prevents angina pectoris and myocardial infarction. Therefore, the decreased use of fibrate did not affect the outcome. The frequency of use of statins, calcium antagonists, and angiotensin-II receptor blockers may be similar to that of patients being treated for dyslipidemia or hypertension. Regarding risk factors, comprehensive treatment with these medications might have ameliorated the arteriosclerotic outcomes in the patients with Werner syndrome.

Although the percentage of patients with intractable skin ulcers decreased compared to that reported a decade ago, two thirds of patients with Werner syndrome still had intractable ulcers. The nationwide survey revealed that a high percentage of patients with Werner syndrome were reported by plastic surgeons or dermatology specialties. We speculate that patients with Werner syndrome visited the hospital for the treatment of ulcers or to receive more specialized treatments.

The percentage of patients with soft-tissue calcification increased. Soft-tissue calcification was changed from “other symptoms” to “major symptoms” in the 2012 diagnostic criteria. Therefore, soft-tissue calcification may have been checked more frequently than in 2009.

The registry contributed to the recruitment of patients with Werner syndrome for a clinical trial. Based on the Werner Registry, patients were introduced to a physician-initiated clinical study of limb ulcers treated with the functional peptide, SR-0379. Treatment with this peptide resulted in reduced size of skin ulcers compared with a placebo after 28 days [19, 20]. The reduction rate of ulcer size in patients with Werner syndrome treated with 0.1% SR-0379 was 22.90%. The DESIGN-R score index, which is calculated based on six components (exudate, size, infection/inflammation, granulation tissue, necrotic tissue, and pocket size) and used as a tool to score pressure ulcer severity, decreased by 4.0 points in patients with Werner syndrome [20]. In near future, this functional peptide may be able to use as the treatment for the patients with Werner syndrome and intractable skin ulcers. As results, it may lead to reduce limb amputation.

The physique of patients with Werner syndrome is smaller than that of the average Japanese in the fifth decade of life. Moreover, the SMI of the patients was far below the threshold of 7.0 kg/m^2^ for men and 5.4 kg/m^2^ for women, which is one of the diagnostic criteria for sarcopenia in Asia according to the Asian Working Group for Sarcopenia [21]. Handgrip strength, another diagnostic criteria for Asian sarcopenia, was well below the thresholds of <28 kg and <18 kg for men and women, respectively [21]. Another indicator of sarcopenia, walking speed, was <1.0 m/sec on average, and most patients performed below the threshold. Therefore, in this registry, most patients aged over 40 years had sarcopenia. At least half of patients also had visceral fat accumulation; therefore, they have exhibited lipodystrophy and sarcopenic obesity. Reportedly, the observed prevalence of sarcopenia in patients with type 2 diabetes aged 65 years and older has been reported to be 18.7% in Japanese outpatient clinics [22]. Therefore, the prevalence of sarcopenia and sarcopenic obesity in patients with Werner syndrome is higher than that in patients with type 2 diabetes aged >65 years. Patients with sarcopenic obesity are generally less active and are at a higher risk of falls, fractures, and death [23–26].

Almost all patients showed a decrease in grip strength; however, two patients did not show a decrease in grip strength. One of these patients belonged to the Self-Defense Forces in his twenties. His muscle training exercises and well-balanced diet may have affected his grip strength result. Therefore, muscle training and nutrition improvement may be useful for preventing sarcopenia. Sarcopenia appears early in most patients with Werner syndrome; therefore, sarcopenia in the patients Werner syndrome may be prevented by early intervention with strength training and with treatments that include amino acids such as leucine, whey protein, calcium, and vitamin D [27, 28].

Fortunately, the percentage of patients with malignant tumors has decreased compared with the percentage in 2009. However, morbidity of malignant tumors is still high in patients with Werner syndrome. Reportedly, the age at cancer diagnosis in patients with Werner syndrome had advanced by 20 years compared with that in the general Japanese population [29]. Therefore, periodical cancer screening is required. Reportedly, Werner syndrome patients with diabetes had a significantly higher cancer prevalence than Werner syndrome patients without diabetes [30]. Therefore, especially for Werner syndrome patients with diabetes, periodic cancer screening is important.

There was a report that dementia and/or schizophrenia appears around 40 years of age [8]; however, there were no patients with dementia and/or schizophrenia identified in this registry.

We plan to maintain the registry active and conduct a longitudinal analysis. We also intend to use the data obtained by the registry follow-up as evidence for the revision of Werner syndrome clinical practice guidelines. We are also developing tools that can be used for early diagnosis based on the characteristics of the voice and face. We are planning clinical trials to examine treatment with new medicines, such as nicotinamide riboside.

Some limitations of the study should be noted. Since Werner syndrome is a rare disease, the number of patients was small. Of the patients diagnosed with Werner syndrome in the nationwide survey, 34.5% were included in this registry; therefore, this is one of the largest databases of patients with Werner syndrome. We continue to enroll more patients in this registry to make it more complete. The questionnaires of the registry require to be improved in the following aspects: The effects of drug prescriptions were not evaluated and further studies should address effective treatments in this patient population. Osteoporosis was not included in the survey items and should be added to the survey items in future. The false positive/negative rates also require to be evaluated.

In conclusion, the results of the Werner Syndrome Registry revealed the current disease profile of patients with Werner syndrome in Japan. The data suggest that although the prognosis of patients with Werner syndrome has not worsened, there are opportunities for early diagnosis and intervention, which may result in improved quality of medical treatment of patients with Werner syndrome. Sensitization about this condition is needed.

## MATERIALS AND METHODS

### The nationwide survey

A nationwide primary survey was conducted to identify patients who were diagnosed with Werner syndrome, in a collaboration with the National Health Labor Science Research Policy Research Project. Primary information of Werner syndrome patients was also gathered and updated, based on the results of the previous nationwide survey that was conducted in 2009 [7]. For the nationwide survey, questionnaires were sent to 7888 physicians in Japan in 2017. The physicians were asked whether they had patients with Werner syndrome based on the diagnostic criteria [7]. The difference in patients’ distribution for each region was analyzed using Wilcoxon rank sum test. JMP pro 13 (SAS Institute, Cary, NC) was used in the analysis.

### Establishment, management, data collection and analysis of the Werner Syndrome Registry

The Werner Syndrome Registry was established to investigate the disease, recruit participants for clinical trials, and to provide information to enrolled patients and physicians. A data sheet for the registration system (Supplementary File) was prepared, based on the previous survey [7], and was referenced to domestic and international intractable disease registration systems. For the Werner Syndrome Registration system, DATATRACK ONE (NTT DATA, Tokyo, Japan) has been used, supported by the Chiba University Clinical Research Center. A registry infrastructure has been completed.

The facilities that reported definitive Werner syndrome cases in the nationwide survey participated in the registry and performed case registration of confirmed diagnosed patients. We obtained informed consent to access the survey of case information, in which the data of clinical symptoms/natural history (course from onset to treatment start), mutation pattern of the causative gene, and treatment information were collected. Blood samples were collected from the patients who provided consent, which served as a repository; complete blood count, liver function, renal function, electrolytes, lipid profile, and glucose metabolism were measured. As patients’ background, age at enrolment, onset age, and age at diagnosis were investigated. As physical findings, height, body weight, and BMI, waist circumference, visceral fat area measured by computed tomography, and SMI measured by DEXA were investigated. As physical function, mean grip strength and mean walking speed were investigated. The patients’ data were collected annually to enable cross-sectional and longitudinal analysis.

Data at enrollment for each patient of the registry was analyzed with JMP pro 13 (SAS Institute, Cary, NC), and served as the pilot data. The data were extracted on July 6, 2020. Regarding major signs, the clinical symptoms, and treatments, the data in this registry were compared with the data in 2009. Fisher’s exact test was used for the comparison.

The study complied with the ethical rules for human experimentation as specified by in the Declaration of Helsinki. The study received approval from the Ethics Board of Chiba University on 27^th^ July 2016, approval number 278. The study was registered at UMIN Clinical Trial Registry (https://upload.umin.ac.jp/cgi-open-bin/ctr_e/ctr_view.cgi?recptno=R000034058) on 3^rd^ November 2017 (ID: UMIN000029812).

The key inclusion criteria for the registry were as follows: 1) patients with confirmed Werner syndrome based on the diagnostic criteria [7] and 2) patients who provided written informed consent prior to their participation in the study.

## Supplementary Material

Supplementary Figure 1

Supplementary Tables

Supplementary File 1
