# Supplementary material for: Time gap between the onset and diagnosis in Werner syndrome: a nationwide survey and the 2020 registry in Japan
Source: Aging (Albany NY). 2020 Dec 29;12(24):24940–56. doi: 10.18632/aging.202441 (PMC7803551; doi:10.18632/aging.202441)
Supplement: Supplementary Figure 1 [file aging-12-202441-s001.pdf]

SUPPLEMENTARY FIGURE

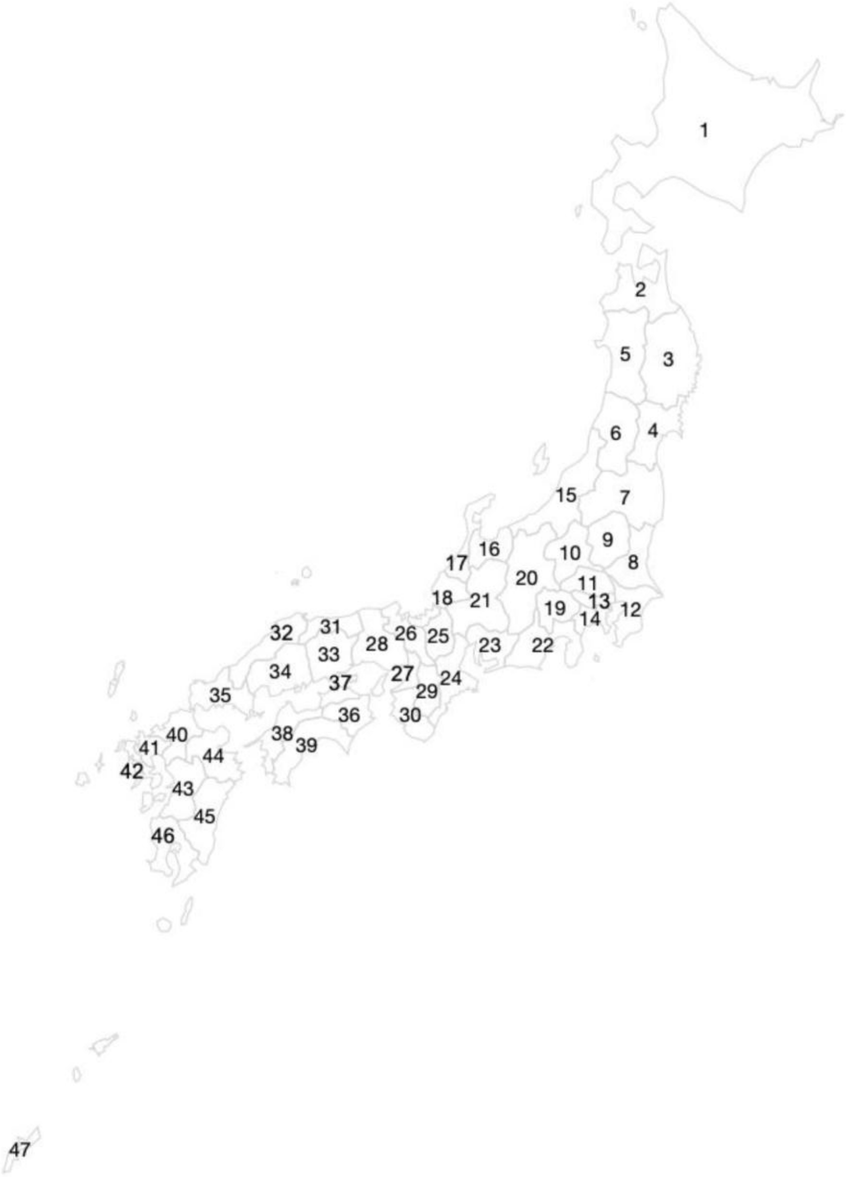

Supplementary Figure 1. Number of patients with Werner syndrome in each region in Japan. Number on map indicates in Supplementary Table 2.
