# Supplementary material for: Time gap between the onset and diagnosis in Werner syndrome: a nationwide survey and the 2020 registry in Japan
Source: Aging (Albany NY). 2020 Dec 29;12(24):24940–56. doi: 10.18632/aging.202441 (PMC7803551; doi:10.18632/aging.202441)
Supplement: Supplementary Tables [file aging-12-202441-s002.pdf]

SUPPLEMENTARY TABLES

Supplementary Table 1. Breakdown of patients with Werner syndrome and suspected cases.

	Total	Male	Female
Patients with Werner syndrome at the hospitals at the time of the survey	116	57	59
Patients suspected of having Werner syndrome	51	29	22
Patients with Werner syndrome visited the hospital in the past 10 years	153 (The sexes of two patients were unknown)	80	71
Total	320	166	152

Reported patients includes 116 diagnosed patients who were attending the hospital for treatment during the survey, 51 patients suspected of having Werner syndrome, and 153 patients visited the hospital in the past 10 years although not having attended the hospitals during the survey. In total, 320 patients with Werner syndrome or suspected cases were reported.

Supplementary Table 2. Number of patients with Werner syndrome in each region in Japan.

Region	Number on map	Diagnosed cases	Suspected cases	Past confirmed cases	Combination	Diagnosed cases / million	Combination / million	Population (million)
Nagasaki	42	8	0	8	16	5.9	11.8	1.35
Tokushima	36	4	1	2	7	5.4	9.4	0.74
Akita	5	5	0	0	5	5.0	5.0	1.00
Saga	41	3	0	2	5	3.6	6.1	0.82
Kyoto	26	7	1	7	15	2.7	5.8	2.60
Okayama	33	5	2	4	11	2.6	5.8	1.91
Oita	44	3	1	2	6	2.6	5.2	1.15
Yamanashi	19	2	1	1	4	2.4	4.9	0.82
Nara	29	3	0	1	4	2.2	3.0	1.35
Ibaraki	8	4	2	5	11	1.4	3.8	2.89
Niigata	15	3	0	0	3	1.3	1.3	2.27
Miyagi	4	3	3	2	8	1.3	3.4	2.32
Fukui	18	1	1	1	3	1.3	3.9	0.78
Osaka	27	10	3	14	27	1.1	3.1	8.82
Kumamoto	43	2	3	2	7	1.1	4.0	1.77
Mie	24	2	1	1	4	1.1	2.2	1.80
Hyogo	28	6	0	4	10	1.1	1.8	5.50
Aichi	23	8	5	3	16	1.1	2.1	7.53
Gunma	10	2	1	6	9	1.0	4.6	1.96
Toyama	16	1	1	0	2	0.9	1.9	1.06
Miyazaki	45	1	0	2	3	0.9	2.8	1.09
Ishikawa	17	1	1	5	7	0.9	6.1	1.15
Shizuoka	22	3	1	1	5	0.8	1.4	3.68
Iwate	3	1	1	1	3	0.8	2.4	1.26
Ehime	38	1	0	1	2	0.7	1.5	1.36
Hokkaido	1	3	1	6	10	0.6	1.9	5.32
Gifu	21	1	0	1	2	0.5	1.0	2.01
Nagano	20	1	2	4	7	0.5	3.4	2.08
Chiba	12	3	1	2	6	0.5	1.0	6.25
Tokyo	13	5	6	11	22	0.4	1.6	13.72

Hiroshima	34	1	0	4	5	0.4	1.8	2.83
Fukuoka	40	1	3	8	12	0.2	2.3	5.11
Kanagawa	14	1	1	6	8	0.1	0.9	9.16
Okinawa	47	0	1	0	1	0	0.7	1.44
Saitama	11	0	1	2	3	0	0.4	7.31
Yamagata	6	0	1	2	3	0	2.7	1.10
Yamaguchi	35	0	2	1	3	0	2.2	1.38
Shiga	25	0	0	1	1	0	0.7	1.41
Kagoshima	46	0	0	2	2	0	1.2	1.63
Aomori	2	0	1	2	3	0	2.3	1.28
Tottori	31	0	0	1	1	0	1.8	0.57
Shimane	32	0	0	1	1	0	1.5	0.69
Tochigi	9	0	2	3	5	0	2.6	1.96
Fukushima	7	0	0	3	3	0	1.6	1.88

Number on map indicates in Supplementary Figure 1.
