# Supplementary material for: Time gap between the onset and diagnosis in Werner syndrome: a nationwide survey and the 2020 registry in Japan
Source: Aging (Albany NY). 2020 Dec 29;12(24):24940–56. doi: 10.18632/aging.202441 (PMC7803551; doi:10.18632/aging.202441)
Supplement: Supplementary File 1 [file aging-12-202441-s003.docx]

Werner Syndrome Registry Sheet

Date of description：year , month , day

Last consultation date：year , month , day

【Patient information】

Registration identification ID :

Sex : 　　Male　　/ 　Female

Date of birth : year , month , day Age : 　　　　　　year

Date of onset : year , month , day Diagnosis date : year , month , day

【Body findings】

Height : 　　　　cm 　　Weight : 　　　　kg　　BMI : 　　　　kg/m^2^

Umbilical abdominal circumference : 　　　　　cm

Blood pressure : / mmHg　 Pulse : bpm　regular・irregular

Amputation of limbs :　　　　　　　　　　　　　　　　　Yes　　/ No

(If Yes, Site : Surgery date : year , month , day )

【Major signs】

Graying of hair, hair loss :　　　　 　　　Yes　　/ No

Cataracts (bilateral) :　　　　　　　　　　　　　 　　　Yes　　/ No

Skin atrophy / hardening : 　　　Yes　　/ No

Intractable skin ulcers :　　　　　　　　　　　　 　　　Yes　　/ No

Soft-tissue calcification : 　　　Yes　　/ No

Bird-like face :　　　　　　　　　　　 　 　　　　Yes　　/ No

High-pitched voice :　　　　　　　　　 　 　　　　Yes　　/ No

【Other signs】

Diabetes, impaired glucose tolerance :　　 　　　　 　　　Yes　　/ No

Dyslipidemia :　　　　　　　　　　　　　　　　 　　　Yes　　/ No

Hypertension :　　　　　　　　　　　　　　　　 　　　Yes　　/ No

Fatty liver :　　　　　　　　　　　　　　　　　 　　　Yes　　/ No

Cerebral bleeding :　　　　　　　　　　　　　　 　　　Yes　　/ No

Cerebral infarction :　　　　　　　　　　　　　　 　　　Yes　　/ No

Angina pectoris or myocardial infarction :　　　　　 　　　Yes　　/ No

Arteriosclerosis obliterans :　　　　　　　　　 　　　Yes　　/ No

　 Malignant tumor :　　　　　　　　　　　　　 　　　Yes　　/ No

　　(If Yes, Name of Disease : )

　　Consanguineous marriage :　　　　　　　　　　　　　　Yes　　/ No

　　Genetic test :　　　　　　　　　　　　　 　　　Yes　　/ No

　　(If Yes, Variant type : Inspection date : year , month , day )

【Blood test findings】（Date : year , month , day ）

(Blood cell count)

WBC : 　　　 /μL,　RBC :　　 ×10^4^/μL,　Hb : 　　　g/dL, Plt : 　　　×10^4^/μL,

(Liver function)

AST : 　　　IU/L, ALT : 　　　IU/L,　γ-GTP : 　　　IU/L, LDH : 　 　　IU/L,

ALP : 　 　 IU/L,　ChE : 　　　　IU/L, T-Bil : 　　　mg/dL,

(Lipids)

T-Cho : 　 mg/dL, TG : 　　 mg/dL, LDL-C : 　 mg/dL, HDL-C : mg/dL,

(Biochemistry)

TP : g/dL, Alb : g/dL,

(Renal function)

UA : mg/dL, BUN : mg/dL, Cre : mg/dL,

(Electrolytes)

Na : mEq/L, K : mEq/L, Cl : mEq/L, Ca : mg/dL,

(Glucose)

　　　Blood glucose (Fasting・Non-fasting) : 　　　mg/dL, HbA1c (NGSP) : 　　　%

【Body composition】

Umbilical visceral fat area (CT) : 　　　　　cm^2^ Date : year , month , day

Lean body mass of limbs : 　　　　　　kg SMI : 　　　　　　　kg/m^2^

Date : year , month , day

【Physical function test】

Grip strength : Right　　　　　kg / Left　　　　　kg　 Date : year , month , day

Walking speed : 　　　　　m/sec　Date : year , month , day

【Oral medicine】

【Oral medicine】

(Diabetes drug)

　　DPP4 inhibitor : 　Yes　／　No

　　Biguanide : 　Yes　／　No

　　Thiazolidine : 　Yes　／　No

　　Alpha-glucosidase inhibitor : 　Yes　／　No

　　Sulfonyl urea : 　Yes　／　No

　　Glinide : 　Yes　／　No

　　SGLT2 inhibitor : 　Yes　／　No

　　GLP-1RA : 　Yes　／　No

　　Insulin : 　Yes　／　No

　　Others : 　Yes　／　No　(If Yes : Drug name　　　　　　　　　　　　　　)

(Dyslipidemia drug)

　　Statins : 　Yes　／　No

　　Fibrate : 　Yes　／　No

　　Ezetimibe : 　Yes　／　No

　　EPA : 　Yes　／　No

　　Ion-exchange resin : 　Yes　／　No

　　Nicotinic acid : 　Yes　／　No

　　Others : 　Yes　／　No　(If Yes : Drug name　　　　　　　　　　　　　　)

　(Antihypertensive drug / drug for heart disease)

　　Calcium channel blocker : 　Yes　／　No

　　Angiotensin receptor antagonist: : 　Yes　／　No

　　ACE inhibitor : 　Yes　／　No

　　Alpha 1 receptor antagonist : 　Yes　／　No

　　Beta receptor antagonist : 　Yes　／　No

　　Diuretics : 　Yes　／　No　(If Yes : Drug name　　　　　　　　　　　　　　)

　　Eplerenone : 　Yes　／　No

　　Nitrate : 　Yes　／　No

　　Aspirin : 　Yes　／　No

　　Antiplatelet drug (other than aspirin) : 　Yes　／　No

⇒ (If Yes : Drug name　　　　　　　　　　　　　　)

　　Warfarin : 　Yes　／　No

　　Anticoagulant (other than warfarin) : 　Yes　／　No

⇒ (If Yes : Drug name　　　　　　　　　　　　　　)

　　Others : 　Yes　／　No　(If Yes : Drug name　　　　　　　　　　　　　　)

【Others】 (Remarks column)

【Name of institute】

【Name of doctor】
